# Supplementary material for: Effective Multivalent Oriented Presentation of Meningococcal NadA Antigen Trimers by Self-Assembling Ferritin Nanoparticles
Source: Int J Mol Sci. 2023 Mar 24;24(7):6183. doi: 10.3390/ijms24076183 (PMC10093968; doi:10.3390/ijms24076183)
Supplement: Supplementary file 1 [file ijms-24-06183-s001.zip › ijms-2274268-supplementary.pdf]

Article

Effective Multivalent Oriented Presentation of Meningococcal NadA Antigen Trimers by Self-Assembling Ferritin Nanoparticles

Daniele Veggi [1,†], Lucia Dello Iacono [1,†], Enrico Malito [2,‡], Giulietta Maruggi [2], Fabiola Giusti [1], Panchali Goswami [3], Werner Pansegrau [1], Sara Marchi [1], Sara Tomei [1], Enrico Luzzi [1], Matthew James Bottomley [2,§], Federico Fontani [1], Ilaria Ferlenghi [1] and Maria Scarselli [1,*]

[1] GSK, Via Fiorentina 1, 53100 Siena, Italy
[2] GSK, 14200 Shady Grove Rd, Rockville, MD, USA
[3] GSK, Gunnels Wood Rd, Stevenage SG1 2NY, UK
* Correspondence: maria.x.scarselli@gsk.com
† These authors contributed equally to the work.
‡ Current address: Dynavax Technologies, 2100 Powell Street, Suite 720, Emeryville, CA 94608, USA.
§ Current address: Structural Biology at Schrödinger, 1540 Broadway, New York, NY 10036, USA.

Supplementary Materials

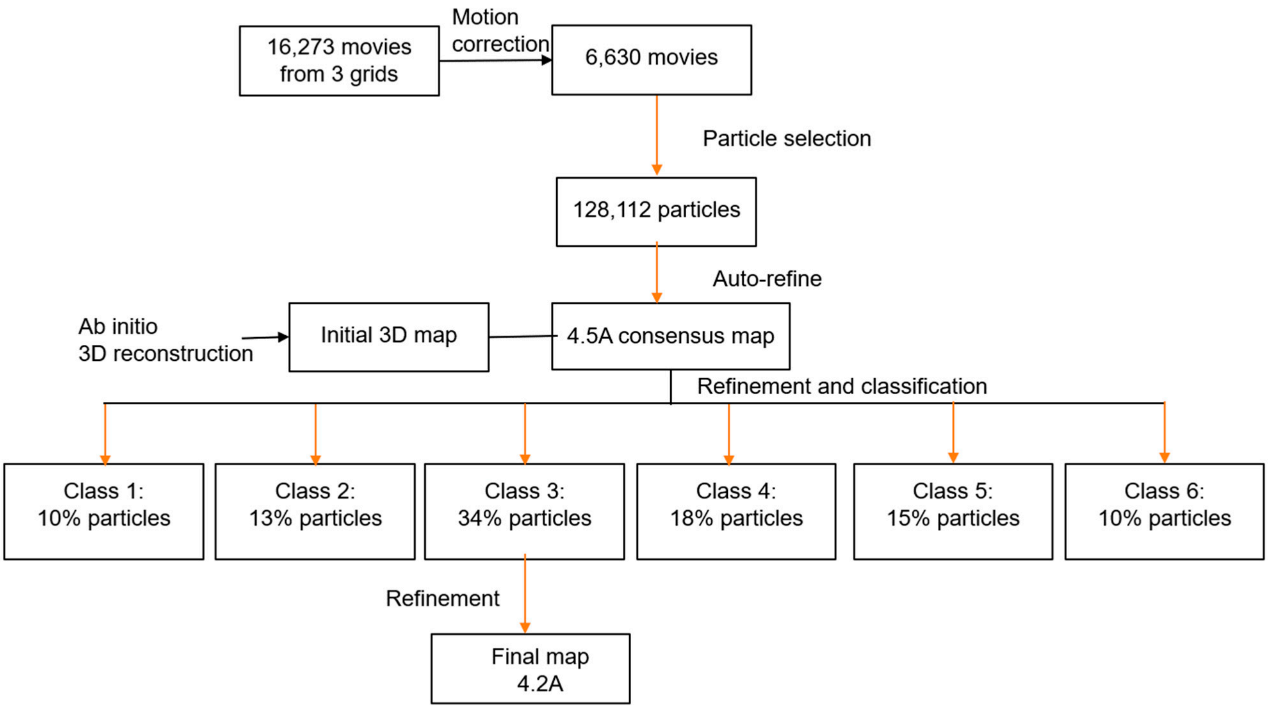

Figure S1. Flowchart for cryo-EM data processing of the NadA_Sht-Fe. Overview of all steps and blocks from the corresponding processing generated by running Relion 3.0 (Zivanov J 2018). All steps are automated.

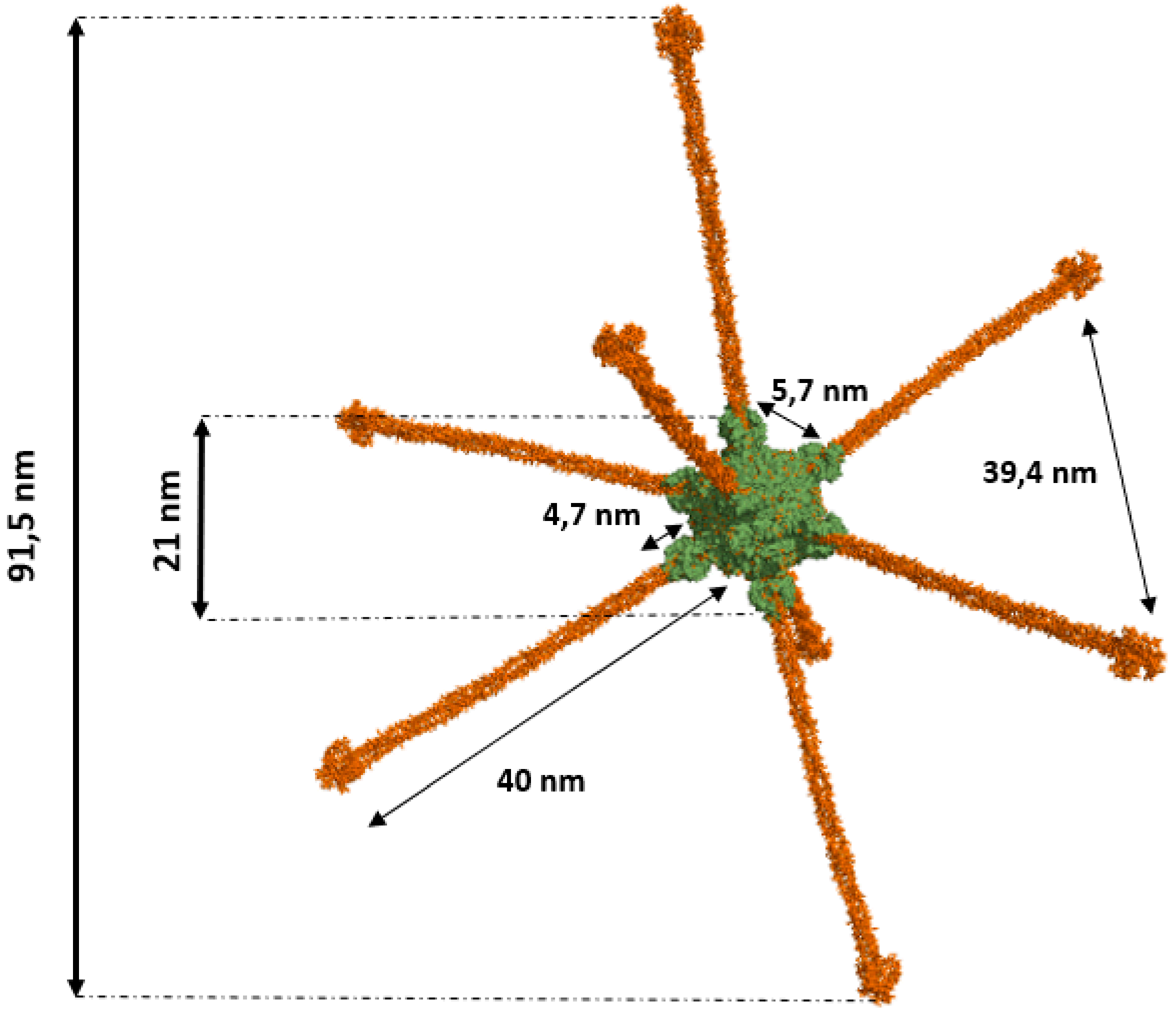

Figure S2. Superimposed computer models of NadA_Sht-Fe (green) and NadA_Ext-Fe (brown). X-ray coordinates of *Helicobacter pylori* ferritin (PDB code 3EGM) were joined at the N-terminus with NadA 24-89 or NadA 24-345, preceded by the SGGAGS linker. NadA coordinates were derived from the PDB entry 6EUN, except for the region 170-235 that was modelled ex novo as a trimeric coiled coil helix.

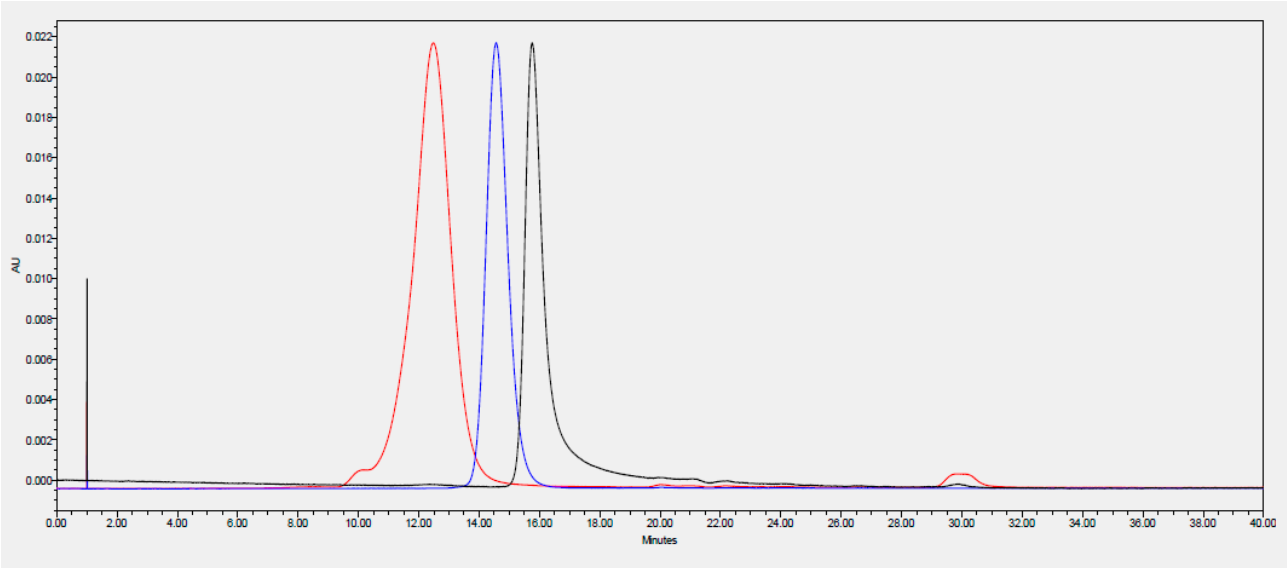

Figure S3. Size Exclusion chromatography profile of different NadA-ferritin and ferritin-alone nanoparticles. NadA_Ext-Fe (red), NadA_Sht-Fe (blue), and ferritin (black).

A

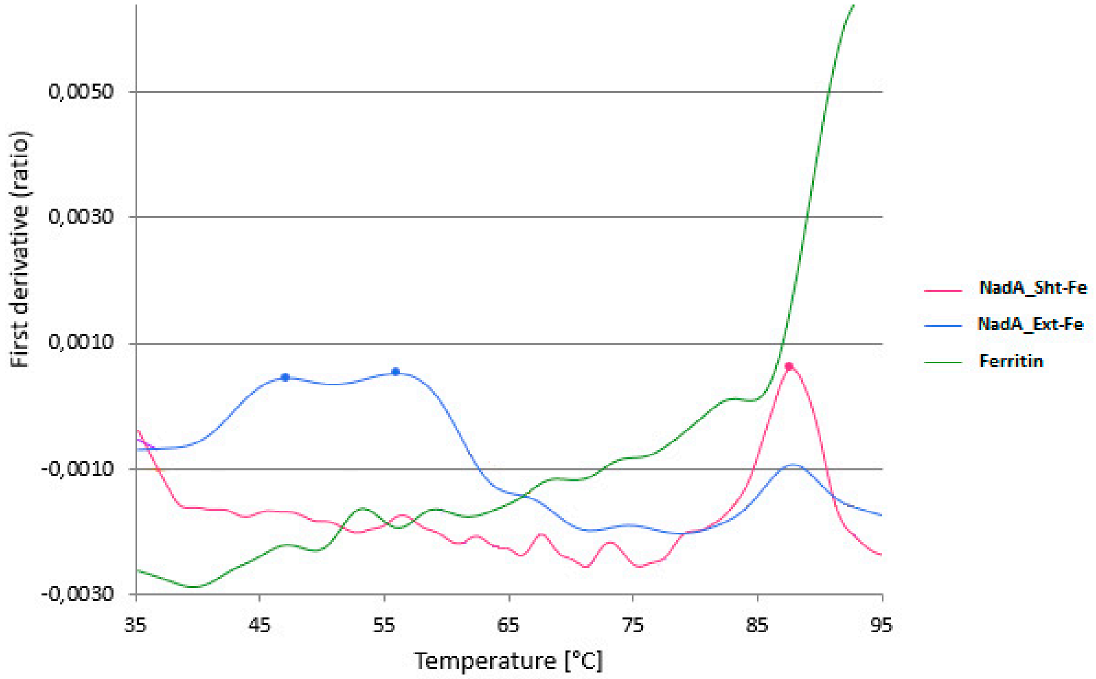

B

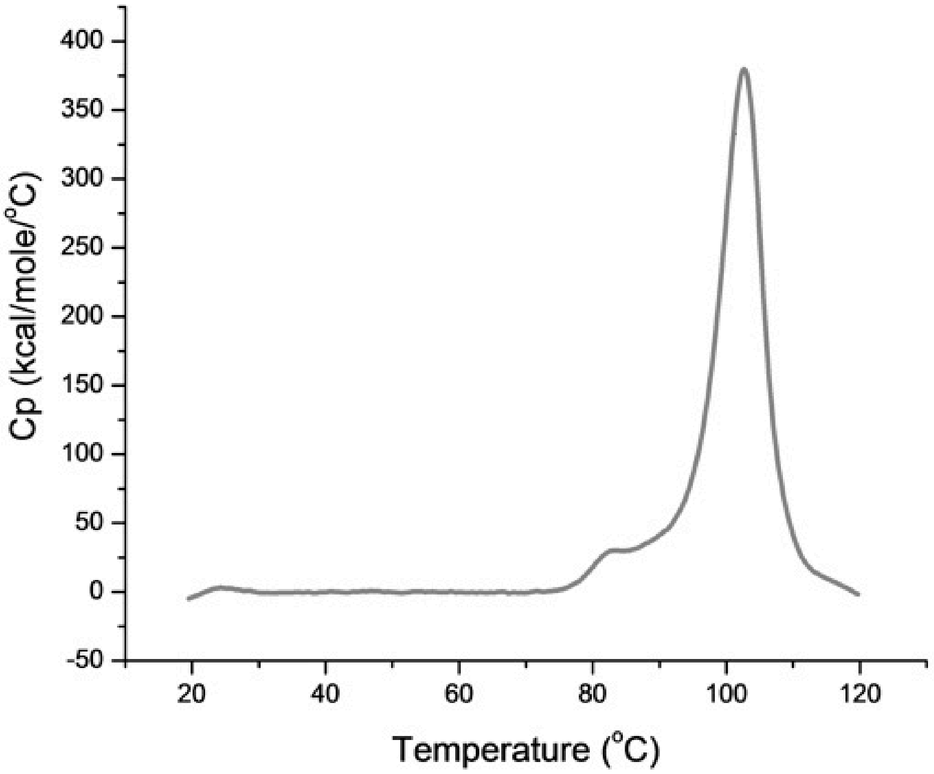

Figure S4. Thermostabilty of NadA_Sht-Fe and NadA_Ext-Fe constructs. (**A**) = DSF profiles (**B**) = DSC thermogram of uncoupled ferritin.
